# Psychometric Properties of the Participation Motivation Questionnaire Among Hungarian Female Students

**DOI:** 10.1002/hsr2.72696

**Published:** 2026-06-20

**Authors:** Narges Ghazvini, Pál Hamar, Johanna Takács, Leonidas Petridis, István Soós

**Affiliations:** ^1^ Doctoral School, Hungarian University of Sports Science Budapest Hungary; ^2^ Department of Pedagogy Hungarian University of Sports Science Budapest Hungary; ^3^ Sapientia Hungarian University of Transylvania Sf. Gheorghe Romania; ^4^ Ludovika University of Public Service Budapest Hungary; ^5^ Department of Social Sciences Semmelweis University Budapest Hungary; ^6^ Research Center for Sport Physiology Hungarian University of Sport Science Budapest Hungary

**Keywords:** Hungarian female students, participation motivation questionnaire, physical activity, self‐determination theory, sociodemographic factors

## Abstract

**Background and Aim:**

Based on the self‐determination theory (SDT), this study primarily aimed to examine the reliability and validity of the Hungarian version of the Participation Motivation Questionnaire (PMQ) among 13–15‐year‐old female students, and secondarily to describe their motivational profiles and the relationships between motives and physical activity level.

**Methods:**

Three hundred twenty‐five female students (mean age = 14.07; SD = 0.87) from nine different schools—wealthy, moderate, and lower income— were randomly sampled from Budapest and its suburbs. Exploratory Factor Analysis was used with the maximum likelihood extraction method and varimax rotation. To establish construct validity, Confirmatory Factor Analysis was conducted. Internal consistency of the questionnaire and its subscales was assessed using Cronbach's alpha.

**Results:**

A 27‑item, six‐factor solution emerged, partially differing from the original eight‐factor structure, and showed acceptable internal consistency (*α* = 0.73–0.84). Associations between motivational factors and sociodemographic characteristics (age, socioeconomic status, urban–rural residence) and physical activity level revealed significant effects of sociodemographic factors on motivation. Fitness emerged as the only motivational factor showing a strong, direct relationship with adolescents' physical activity level. In terms of SDT, intrinsic‑oriented, health‑ and mastery‑related reasons appeared more important than ego‑related and extrinsic reasons for encouraging female students to be physically active. Given the cross‐sectional design and the urban‐skewed sample, these subgroup findings should be interpreted with caution and considered as a means of generating hypotheses.

**Conclusion:**

In conclusion, the Hungarian version of the PMQ appears suitable for assessing participation motives among female students in school and sport‐related settings and may support educators and practitioners in better understanding motivational patterns when planning and evaluating physical activity programmes.

## 1 Introduction

Despite the well‐known benefits of regular physical activity (PA) (e.g. lower risk of non‐communicable diseases, higher physical and cognitive performance, enhanced mental well‐being and weight control) [1], 81% of adolescents, aged 11 to 17 years were physically inactive globally [2]. The rate of physical inactivity is more profound in adolescent girls with 85% of girls failing to achieve the World Health Organization's (WHO) guideline of at least 60 min of moderate to vigorous physical activity daily, compared to 78% of boys (WHO, 2022). Hungarian adolescents are no exception; only 41% of children and adolescents in Hungary aged 5 to 17 years meet the minimum guidelines recommended by the World Health Organization [3]. The Organization for Economic Co‐operation and Development (OECD) identifies Hungary as one of the European countries with a high obesity rate. The prevalence of overweight and obesity among adolescents in Hungary is claimed to be 25%, ranking Hungary with the fourth highest rate in the European Union [4]. Within this context, it is important to understand the motivations of adolescent girls for engaging in or disengaging from physical activity to design targeted, cost‐effective interventions and inform school‐ and community‐level health policies [5].

Numerous psychological characteristics have been recognized as causative determinants of physical activity involvement with motivation being a major component in the realm of sport and physical activity [6]. Deci and Ryan's Self‐Determination Theory (SDT) is a cognitive framework that effectively defines the motivational determinants and perseverance in physical activity. The SDT posits that three fundamental demands drive individual motivation: autonomy, competence, and relatedness [7, 8]. Furthermore, the need for autonomy and competence underpins the intrinsic‐extrinsic motivation dichotomy, which has been extensively examined in the sport and exercise psychology literature [7, 8]. An individual's propensity to engage in exercise is contingent upon their intrinsic and extrinsic motivations, or on the nature of their goals. Diverse participation motives may have distinct functional meanings depending on their intrinsic–extrinsic motivational alignment [9]. Vallerand [10] posits that intrinsic motivation in sport and physical activities derives its driving forces from personal delight or satisfaction derived directly from participation itself, while extrinsic motivation pertains to engagement driven by external factors such as rewards, recognition, or acceptance from society. The motivational patterns are not only theoretically relevant but also directly linked to behavior change, adherence to physical activity guidelines, and long‐term prevention of noncommunicable diseases. Reliable assessment of adolescents' motives, therefore, represents a practical tool for identifying at‐risk groups, evaluating the effectiveness of school‐based and community programs, and increasing long‐term healthy habits [11].

In the research of Li [12], among Chinese middle school students, sports friendship quality can predict mental health, sport motivation, and sports adherence positively. In research among Turkish participants, those who have high well‐being levels were found to have a strong motivation for participating in sports [13]. A study by Del Pilar Vílchez and De Francisco [14] on Spanish participants showed that females practice exercise and sports for competition and teamwork more than males. In the study of Saarinen et al. [15], the results showed that girls had a stronger mastery orientation both in the sporting and academic domains than boys in Finnish sports high schools. Therefore, these findings indicate that motivation for sport and physical activity is shaped by gender, social background, and cultural context and that they are meaningfully associated with the health field and sustained participation [16]. However, evidence from the Visegrád countries (Czech Republic, Hungary, Poland, Slovakia) has shown that a considerable percentage of adolescents, especially girls, did not meet the recommendations for physical activity, and this is an area that requires further investigation [17].

One of the most popular scales to assess motivation for physical activity and sport is the Participation Motivation Questionnaire (PMQ) [18]. The PMQ is a 30‐item survey that defines some possible reasons for engaging in sport and is answered based on a 5‐point Likert scale. This questionnaire provided a somewhat equitable comparison across numerous studies over several years [14, 19, 20, 21]. Regrettably, despite extensive usage and translation into numerous languages, certain criteria lack psychometric evidence due to inadequate internal consistency [20, 22, 23]. Considering individuals from different societies participate in physical and sports activities for various reasons, validating and adapting the PMQ is therefore not a purely methodological exercise; it is a prerequisite for using motivation data to guide targeted health promotion, monitor intervention outcomes, and support evidence‑informed policy decisions in school and community settings. Given the 81% prevalence of physical inactivity among adolescent girls, it is important to note that the PMQ has not been validated in the Hungarian population, and no studies have examined its psychometric properties specifically among Hungarian female students. Moreover, there is limited evidence on how different motives for participation relate to sociodemographic factors and physical activity levels in this group, which restricts the ability of educators, coaches, and health professionals to design context‑specific interventions. Therefore, this study aimed to investigate the reliability and validity of the PMQ and, secondly, the motivational profile of 13–15‐year‐old female students attending schools in Hungary. In addition, this study aimed to examine the differences of various motives in relation to age, socioeconomic status (SES), urban or rural residence, and physical activity levels to identify specific subgroups that may benefit from differentiated, equity‑oriented public health and educational interventions.

## 2 Methods

### 2.1 Participants and Procedures

The present study employed a cross‐sectional design and was conducted during the 2022–2023 academic year. Overall, the sample included 325 Hungarian female students aged 13–15 years (*M* = 14.07; SD = 0.87). A two‑stage, school‑based cluster sampling approach was used. First, a list of all state‑funded lower‑secondary schools in Budapest and its suburban belt was obtained from the Ministry of Education and stratified by district‑level socioeconomic indicators (wealthy, moderate‑income, low‑income). Within each stratum, schools were randomly invited until three schools per stratum agreed to participate, yielding nine schools in total. The pupils were requested to evaluate their socioeconomic situation. Socioeconomic status was assessed using parent occupational status. Students were asked to indicate the current occupations of both their mother and father (or primary caregivers). Reported occupations were coded using a standard occupational classification scheme and then grouped into three SES categories (low‐income, moderate‐income, wealthy) [24]. Low‐income SES included manual and unskilled occupations (e.g., factory workers, cleaners, drivers), moderate‐income SES included skilled and lower professional occupations (e.g., technicians, office employees, teachers), and wealthy SES comprised higher professional and managerial positions (e.g., physicians, engineers, senior managers). Based on their responses, 68.9% of the participants were identified as middle‐class, while 21.5% resided in wealthy districts and 9.5% in low‐income districts. Regarding age distribution, 34.8% of the participants were 13 years old, 23.7% were 14 years old, and 41.5% were 15 years old. In terms of residence, 16.3% were from rural areas and 83.7% from urban areas. The sample was therefore heavily skewed towards urban participants, reflecting the metropolitan catchment area of the sampled schools; this imbalance limits the generalizability of the findings to rural Hungarian adolescents and should be borne in mind when interpreting subgroup comparisons by place of residence. According to the International Physical Activity Questionnaire (IPAQ), 13.5% of students were classified as having low physical activity, 28.3% as moderate, and 58.2% as high physical activity levels. After receiving approval from the Hungarian University of Sports Science and the Ministry of Education in Hungary, the questionnaires were made available to volunteer students online with the endorsement of the school principals and consultation with physical education teachers. The Participation Motivation Questionnaire, General Information Demographic Data Questionnaire and IPAQ were completed by students online for approximately half an hour. Additionally, the students had the confidence that their responses would be kept proprietary. For this study, an authorization was issued from the research ethics committee of the Hungarian University of Sports Science, (approval number: TE‐KEB/27/2022). Permission for the completion of all high school students was asked from the parents, and each participating student expressed his or her consent. The study was voluntary, and participants were free to withdraw at any time without explanation.

### 2.2 Measures

#### 2.2.1 Participation Motivation Questionnaire

Motivation was assessed using the Participation Motivation Questionnaire (PMQ) of Gill, Gross and Huddleston [18]. It consists of 30 items with answers given on a 5‐point Likert scale ranging from 1 (not important) to 5 (very important), consisting of 8 sub‐dimensions (achievement/status, physical fitness/working off energy, team membership/spirit, friendship, fun, competition, skill development, and motion/being active). The reliability of this questionnaire, as reported by Zahariadis and Biddle [25], was 0.80. In the present study, the original version of the 30 items of the PMQ was first administered, followed by an Exploratory Factor Analysis to examine the dimensional structure of the PMQ in the target population. Based on the outcomes of the EFA, three items were removed due to low factor loadings (< 0.30), resulting in a 27‑item version of the scale. The remaining 27 items were loaded on six distinct factors: achievement/status, team membership/spirit, being active/skill development, competition/excitement, fitness, and energy release. Thus, the Hungarian version of the PMQ represents a structurally modified form of the original instrument, with a reduced number of items and a more parsimonious six‑factor configuration. The translation of the scale encompassed several phases as suggested previously [26]. The English version of the PMQ was translated into Hungarian by independent experts who specialize in sport psychology and pedagogy. Then, a back‐translation was made by an independent English language specialist to ensure that the original concept would not be lost and that the wording was clear. The team examined the differences between the original text and its back‐translated version to determine which parts needed correction. After reviewing and discussing the translated questionnaire, necessary corrections were made, generating the final draft. A pilot testing with a small group of participants was conducted to verify the items' cultural applicability and comprehension. Feedback from the pilot testing was used to refine item wording where necessary (e.g., simplifying phrases and ensuring age‑appropriate language), thereby strengthening the content validity and cultural adaptation of the Hungarian PMQ.

### 2.3 International Physical Activity Questionnaire (IPAQ)

Physical activity levels were assessed by the short form of the International Physical Activity Questionnaire (IPAQ‐SF), with demonstrated validity and reliability in Hungary [27]. The IPAQ‐SF consists of seven items and records the frequency (days/week) and duration (minutes/day) of vigorous, moderate, and walking activities during the past 7 days. Each activity category was then also multiplied by its standard Metabolic Equivalent of Task (MET) value—8.0 for vigorous, 4.0 for moderate, and 3.3 for walking—and summed to determine total MET‐minutes per week. Participants were then stratified into three ranges based on their total physical activity score: low (< 600 MET‐min/week), moderate (600–3000 MET‐min/week), and vigorous (> 3000 MET‐min/week).

### 2.4 Statistical Analyses

Data processing was performed using the IBM SPSS v. 26 and JAMOVI v. 2.6.26. Descriptive statistics were reported as frequencies, means, and standard deviations. Exploratory Factor Analysis (EFA) was conducted to identify which motivational factors are most important in the target population, classifying the items concerning their factor loadings. The identified subscales at the first stage were further validated by Confirmatory Factor Analysis (CFA) using Structural Equation Modeling (SEM), with consideration of the fit indices criteria (*χ* ^2^(309) = 688, *p *< 0.001; *χ* ^2^(309) = 2.23; CFI = 0.98; TLI = 0.98; SRMR = 0.06; RMSEA = 0.062, 95% CI [0.055, 0.068]). Internal consistency of the subscales was assessed using Cronbach's alpha. Nonparametric tests were conducted because the normality assumptions were not met for the group comparisons of participation motives (*p* < 0.001). The Kruskal–Wallis test was conducted to assess differences by age groups (13, 14, and 15 years), socioeconomic status (wealthy, middle, and low‐income districts), and physical activity levels (low, moderate, and vigorous). The Mann–Whitney U test was also conducted to identify differences between rural and urban participants. The level of significance was set at 0.05.

## 3 Results

To examine the adequacy of the data for EFA, the Kaiser–Meyer–Olkin (KMO) measure and Bartlett's test of sphericity were used. The KMO index value was 0.920, indicating excellent sampling adequacy for factor analysis. In addition, the Bartlett's test of sphericity was significant (*χ* ^2^_(435) _= 4790.395; *p *< 0.01), suggesting that the correlation matrix between the items was suitable for conducting factor analysis. Using the maximum likelihood extraction method and Varimax rotation, six components (factors) with eigenvalues greater than 1 were extracted. These 6 components collectively explained 50.23% of the total variance in the data (Table 1).

**Table 1:** Factor analysis.

Items I am physically active because:	F1	F2	F3	F4	F5	F6
I like to feel important	0.674					
I want to gain status or recognition	0.639					
I want to be popular	0.612					
I like the rewards	0.593					
I like to have fun	0.476					
I like to use the equipment or facilities	0.355					
I like the teamwork		0.803				
I like the team spirit		0.762				
I like being on a team		0.757				
I like to meet new friends		0.396				
I like to have sth to do			0.715			
I like the action			0.600			
I like to get exercise			0.424			
I want to improve my skills			0.422			
I like to do sth I am good at			0.400			
I like to get out of the house			0.313			
I like to compete				0.662		
I want to play sports at a higher level				0.623		
I like the challenge				0.605		
I like to win				0.438		
I want to learn new skills				0.362		
I like the excitement				0.344		
I want to be physically fit					0.633	
I want to stay in shape					0.482	
I like to play sports					0.432	
I want to release tension						0.673
I want to get rid of energy						0.533
Eigenvalues	10.74	1.96	1.70	1.14	1.04	1.04
Variance	10.39	9.74	9.44	9.37	6.08	5.19

Descriptive statistics and correlations of the subscales of the PMQ are presented in Table 2. The greatest mean was found for the Fitness and the lowest for the Achievement/status subscales. Table 2 displays the internal consistency reliability values for the PMQ subscales. The Cronbach alpha coefficients for all six subscales were greater than 0.70. The skewness and kurtosis values for all variables were within the acceptable range (between −2 and +2). All factors, Achievement/status, team membership/spirit, being active/skill development, competition/excitement, fitness, energy release, were significantly and moderately correlated with each other (Table 2).

**Table 2:** Means, standard deviations, subscale correlations, and internal consistencies of the PMQ (*n* = 325).

Variable	Mean	SD	1	2	3	4	5	Alpha
Achievement/status	3.70	0.84						0.81
Team membership/spirit	3.72	1.00	0.52*					0.84
Being active/skill development	4.10	0.69	0.56*	0.60*				0.80
Competition/excitement	3.69	0.87	0.61*	0.57*	0.67*			0.81
Fitness	4.24	0.83	0.46*	0.49*	0.68*	0.63*		0.78
Energy release	3.79	1.06	0.47*	0.39*	0.58*	0.54*	0.50*	0.73

* *p* < 0.01.

The fit indices obtained from the CFA of the six‐factor model of the PMQ (27 items) were predominantly suggestive of an acceptable model. The RMSEA was 0.062, with a 95% confidence interval of 0.055 to 0.068, and a *p* value of 0.001, the SRMR was 0.068, indicating an acceptable level of residual variance, and the normed *χ* ^2^ index (*χ* ^2^(309) = 2.23, *p* < 0.001) was below a maximum of 3.00 [28].

**Figure 1:**
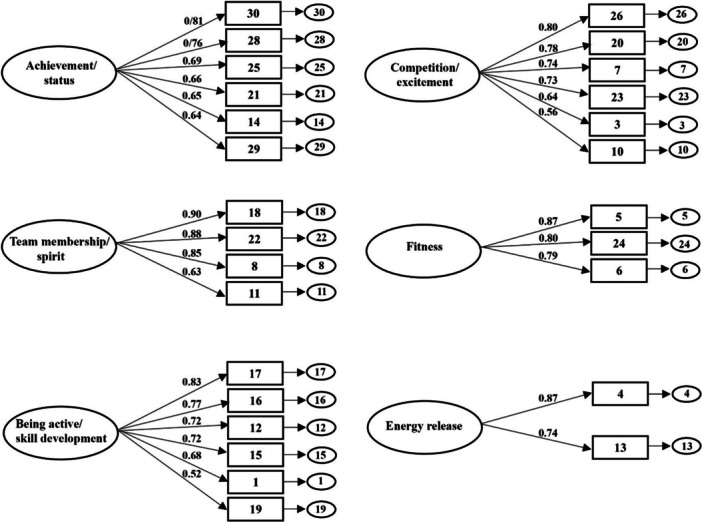
Measurement model (CFA) of the hypothesized six‐factor structure of the PMQ.

The incremental fit indices for this model significantly exceeded the acceptable threshold of 0.90, demonstrating an excellent fit (NFI = 0.97, CFI = 0.98, and TLI = 0.98). The model's parsimony fit was assessed using PNFI. The acquired value of 0.86 is comparatively lower than the incremental fit values, although still within the acceptable range [29]. The six‐factor solution of the PMQ exhibited acceptable item loadings between 0.52 and 0.90, confirming the construct validity of the items, as shown in Figure 1.

**Figure 2:**
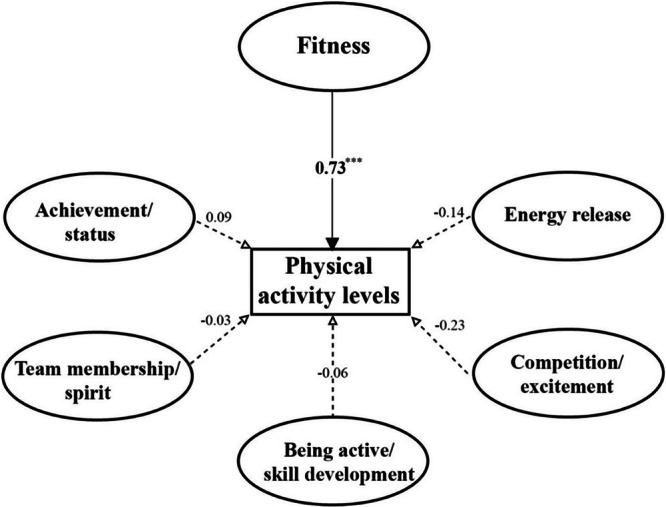
Structural equation model of PMQ dimensions and their associations with physical activity levels.

The CFA analysis results revealed that the model represented in Figure 2 had a satisfactory level of fit to the data (*χ* ^2^(330) = 703, *p* < 0.001; *χ* ^2^(330) = 2.13). The absolute fit measures also satisfied the criteria for goodness of fit: the value of SRMR was 0.06, while that of RMSEA was 0.059 (95% CI [0.053, 0.065]) with a *p* value of 0.007. Additionally, the results of the incremental fit measures clearly depicted that the model had a satisfactory level of fit to the data. This was reflected in high values of NFI (0.97), CFI (0.98), and TLI (0.98) that are all beyond the threshold level of 0.90. The value of the parsimony‐adjusted fit index (PNFI = 0.85) also supported that the level of balance between the goodness of fit and the complexity of the model was satisfactory. With regard to the structural parameters, it was observed that among the studied constructs of participation motivations factors, only fitness had a statistically significant positive effect on physical activity levels as estimated by IPAQ (*β* = 0.73, *p *= 0.001), while the influence of achievement/status, team membership/spirit, being active/skill development, competition/excitement, and energy release was not statistically significant. (Additional details are presented in Supporting Information S1: Table 1).

### 3.1 Differences in Participation Motives According to Sociodemographic Variables and PA Levels

Significant differences were found among several motivational factors according to SES, age, and PA levels, and urban and rural participants. According to SES, the Kruskal–Wallis test revealed significant differences for achievement/status, team membership/spirit, and competition/excitement. Participants from moderate‐income districts reported higher mean ranks in most motivational factors compared to those from wealthy and lower‐income districts. Regarding age, statistically significant differences were observed for all motivational factors except energy release. In general, younger students (13–14 years old) demonstrated higher motivational scores compared to older ones (15 years old). When analyzing PA levels, significant differences were found in all motivational dimensions except Energy release. Participants who had higher levels of PA had higher mean ranks in all these motives. Finally, the Mann–Whitney U test revealed no difference between rural and urban participants for all the motivational factors except Team membership/spirit, on which urban students were more motivated towards teamwork compared to rural students. Although several motivational factors differed significantly by SES, age, PA levels, and urban/rural residence, effect sizes were generally small, with the largest effect observed for Fitness across PA levels. Differences in motivational factors are summarized in Table 3.

**Table 3:** Differences in motivational factors by socioeconomic status, age, physical activity levels, and urban/rural residence.

Motivational factor	SES	Age	PA levels	Urban/rural
Achievement/status	**Moderate > wealthy/lower (*H*(2)** = **8.58, *p*** = **0.014, *ε* ^2^** = **0.02)**	**13–14** > **15 (*H*(2)** = **12.17, *p*** = **0.002, *ε* ^2^** = **0.03)**	**High > low/moderate (*H*(2)** = **10.80, *p*** = **0.005, *ε* ^2^** = **0.03)**	(*Z* = −0.19, *p* = 0.843, *r* = 0.01)
Team membership/spirit	**Moderate > wealthy/lower (*H*(2)** = **6.80, *p*** = **0.033, *ε* ^2^** = **0.02)**	**13** > **14/15 (*H*(2)** = **14.99, *p*** = **0.001, *ε* ^2^** = **0.04)**	**High > low/moderate (*H*(2)** = **6.94, *p*** = **0.031, *ε* ^2^** = **0.02)**	**Urban > rural (*Z*** = **−2.25, *p*** = **0.024, *r*** = **0.19)**
Being active/skill development	(*H*(2) = 2.08, *p* = 0.352, *ε* ^2 ^= 0.00)	**13–14** > **15 (*H*(2)** = **16.18, *p*** < **0.001, *ε* ^2^** = **0.05)**	**High > low/moderate (*H*(2)** = **14.57, *p*** = **0.001, *ε* ^2^** = **0.04)**	(*Z* = −0.12, *p* = 0.899, *r* = 0.01)
Competition/excitement	**Moderate > wealthy/lower (*H*(2)** = **6.81, *p*** = **0.033, *ε* ^2^** = **0.02)**	**13–14** > **15 (*H*(2)** = **17.84, *p*** < **0.001, *ε* ^2^** = **0.05)**	**High > low/moderate (*H*(2)** = **16.14, *p*** < **0.001, *ε* ^2^** = **0.04)**	(*Z* = −1.91, *p* = 0.056, *r* = −0.16)
Fitness	(*H*(2) = 0.85, *p* = 0.654, *ε* ^2 ^= 0.00)	**13–14** > **15 (*H*(2)** = **13.88, *p*** = **0.001, *ε* ^2^** = **0.04)**	**High > low/moderate (*H*(2)** = **34.06, *p*** < **0.001, *ε* ^2^** = **0.10)**	(*Z* = −1.21, *p* = 0.223, *r* = 0.10)
Energy release	(*H*(2) = 0.39, *p* = 0.820, *ε* ^2 ^= 0.00)	(*H*(2) = 5.62, *p* = 0.060, *ε* ^2 ^= 0.01)	(H(2) = 5.92, *p* = 0.052, ε^2^ = 0.01)	(*Z* = −1.45, *p* = 0.146, *r* = 0.12)

*Note:* Statistically significant differences are in bold.

Abbreviations: PA levels, physical activity levels; SES, socioeconomic status.

## 4 Discussion

The WHO emphasizes increasing physical activity levels as a priority for global public health. Validated tools, such as the PMQ questionnaire used in this study, enable researchers and practitioners to understand why some adolescents engage in PA and why others remain inactive. In Hungary, where adolescent overweight and obesity rates are among the highest in the European Union and physical inactivity is highly prevalent, having a psychometrically sound tool that captures why adolescent females do or do not participate in PA is directly relevant to design effective interventions that support healthier lifestyles, reduce the risk of non‐communicable diseases, and tackle the persistently low levels of activity reported, especially among females. Accordingly, the present study not only examined the psychometric properties of the Hungarian version of the PMQ but also generated a motive‑based profile of Hungarian female adolescents that may help inform future school‐, community‐, and policy‐level initiatives. Following exploratory and confirmatory factor analyses, the model comprising six motives demonstrates an appropriate fit to the data. The total explained variance of the six‐factor solution exceeded 50%, which is considered acceptable in psychological and motivational measurement contexts, particularly when examining latent constructs influenced by multiple personal and environmental factors [28]. In addition, the sufficiency of the factor solution was supported by the meaningful interpretability of the factors and the acceptable model fit indices obtained in the confirmatory factor analysis [30]. This six‑factor structure partly diverges from the original eight‑factor PMQ, suggesting a more parsimonious and context‑specific configuration of motives among Hungarian female adolescents. However, despite this divergence, the six factors retained broad coverage of the core motivational domains proposed in the original PMQ, thereby preserving the conceptual comparability of our findings with previous PMQ research. Such structural refinement is important because it indicates which domains of motivation are most salient in this population and which original dimensions (e.g., friendship and fun) may overlap conceptually in this cultural and age group.

The findings of this study align with some of the reasons given within previous research that examined motivation for sport and physical activity participation [14, 19, 20, 23, 31]. According to the Self‐Determination Theory [7], individuals participate in sports and physical activity because of intrinsic motives (such as fun, development, or mastery) and extrinsic motives (such as reward or looking better). In the current study, the highest mean was for the fitness subscale, followed by the being active/skill development subscales, suggesting that Hungarian female students had a stronger intrinsic motivation to engage in physical activity, which is comparable with findings from previous studies [32]; [20, 33, 34, 35, 36]. From the SDT perspective, these results indicate that health‐ and mastery‐oriented, rather than ego‐involving, motives appear to be most relevant to Hungarian female students.

Confirmatory factor analysis of the PMQ demonstrated acceptable item loadings ranging from 0.52 to 0.90, as presented in Figure 1. Given that the same sample was used for both EFA and CFA, these fit indices reflect internal consistency with the exploratively derived structure rather than an independent confirmation.

Generally, internal consistency for all constructs ranged from very good to good, showing high internal consistency and reliability. Although factor intercorrelations were moderate to high, this reflects the theoretically related nature of motivational constructs. Another important aspect in reliability studies is the standard error of measurement, with larger errors denoting lower reliability [30]. The standard errors for most factor loadings of the constructs were relatively small, indicating the stability and accuracy of the estimation of these relationships between the observed and latent variables.

Although the overall model showed satisfactory goodness‐of‐fit indices, fitness emerged as the only motivational variable with a strong, direct association with adolescents' physical activity levels in the present framework (Figure 2). However, given the cross‐sectional design, these associations should be interpreted as correlational rather than predictive. In terms of SDT, the results emphasize that intrinsic‐oriented and health‐ and mastery‐related reasons are more important than ego‐related and extrinsic reasons in encouraging adolescent girls to be physically active.

Our findings revealed that adolescents from the middle class had higher values in most of the sport participation motives compared to those from wealthy and low‐income districts. A systematic review by Gautam et al. [37] verified that adolescents from lower SES have a greater tendency to develop unhealthy lifestyle choices such as physical inactivity. Similarly, in China, physical activity in children and adolescents was found to be associated with SES, such that higher SES was associated with higher levels of physical activity [38]. Adolescents from moderate‐income districts may have adequate access to sport‐related resources while being less pressed by societal or competitive pressures than their counterparts from wealthy‐income districts, which can enhance motivations like team spirit or competition. Yet, these results suggest that health promotion strategies in Hungary should differentiate across different SES, mostly targeting individuals from wealthy and low‐income areas.

The 13–14‐year‐old students reported higher levels of motivation than 15‐year‐olds for achievement/status, team membership/spirit, being active/skill development, competition/excitement, and fitness reasons. For instance, the systematic review conducted by Cachón‐Zagalaz et al. [11] pointed out that adolescence is a critical transition period for developing motivation for physical activity, which is typically influenced by psychosocial and developmental changes.

The positive association between physical activity engagement levels and the mean on all the dimensions of motivation is consistent with motivational theories such as the SDT, suggesting that greater involvement in sport is associated with greater motives.

Based on our investigation, the only dimension that indicated a significant difference relating to urban or rural environment was team membership/spirit, with urban students tending to be more team‐oriented in their motivation. Urbanization and resource inequality have been found to have negative effects on adolescents' physical activity levels, especially in lower socioeconomic areas [39].

## 5 Limitations and Future Goals

Despite the significant findings, the current study exhibited certain limitations. The sample was limited to female pupils aged 13–15 in Budapest; hence, the findings cannot be applied to other age groups, different countries, or rural populations. Secondly, this study utilized the questionnaire developed by Gill et al. [18]. Consequently, it may not include all the motives of participation in sports. The cross‐sectional methodology prevents establishing causal relationships between the identified motivating factors and involvement in physical activity. Six principal motivational factors were identified; however, the particular cultural adaptation method of the questionnaire may have affected the outcomes. Since exploratory and confirmatory factor analyses were conducted on a single sample, to enhance validity, future studies should use an independent sample or split the sample into equivalent subsamples so that confirmatory factor analysis can independently verify the factor structure obtained from exploratory analysis.

## 6 Conclusion

Findings confirm the acceptable validity and adequate reliability of the Hungarian version of the Participation Motivation Questionnaire among Hungarian 13‐15‐year‐old female students. The validity of the six motivational factors identified by confirmatory factor analysis ensures the construct validity of the measure in this specific cultural context. Therefore, the Hungarian version of the questionnaire could be administered safely at school and at sporting facilities for determining the motivational driving factors for physical activity for adolescent female students. Structural equation modeling revealed that fitness was the only strong and direct reason linked to how much physical activity Hungarian adolescent females engage in, highlighting that health and fitness motivations are more important than ego or extrinsic reasons. In addition, sociodemographic factors were found to affect motivational factors significantly. This information may help trainers and teachers design suitable programs commensurate with the needs and wishes of their students, may increase and help to maintain an active lifestyle, and ultimately support better health outcomes in line with the World Health Organization's recommendations for regular physical activity among adolescents.

## Author Contributions

**Narges Ghazvini:** conceptualization, methodology, software, formal analysis, data curation, validation, investigation, resources, visualization, writing – original draft, writing – review and editing. **Pál Hamar:** conceptualization, data curation, investigation, validation, visualization, supervision, project administration, writing – review and editing**. Johanna Takács:** conceptualization, methodology, software, formal analysis, supervision, project administration, visualization, writing – review and editing. **Leonidas Petridis:** conceptualization, methodology, supervision, project administration, visualization, writing – review and editing. **István Soós:** conceptualization, data curation, investigation, validation, supervision, project administration, visualization, writing – review and editing.

## Funding

Open access funding was provided by Doctoral School of the Hungarian University of Sports Science. The funder had no role in the study design, data collection and analysis, decision to publish, or preparation of the manuscript.

## Ethics Statement

The study was conducted according to the guidelines of the Declaration of Helsinki and approved by the Institutional Review Board of the Hungarian University of Sports Science (reference number TE‐KEB/27/2022).

## Consent

Informed consent was obtained from all subjects involved in the study.

## Conflicts of Interest

The authors declare no conflicts of interest.

## Transparency Statement

The corresponding author, Narges Ghazvini, affirms that this manuscript is an honest, accurate, and transparent account of the study being reported; that no important aspects of the study have been omitted; and that any discrepancies from the study as planned (and, if relevant, registered) have been explained.

## Supporting information


**Table S1:** Parameter estimates for paths from PMQ dimensions to physical activity levels.

## Data Availability

The authors confirm that the data supporting the findings of this study are available from the corresponding author upon reasonable request.
