# Supplementary material for: Psychometric Properties of the Participation Motivation Questionnaire Among Hungarian Female Students
Source: Health Sci Rep. 2026 Jun 20;9(6):e72696. doi: 10.1002/hsr2.72696 (PMC13282752; doi:10.1002/hsr2.72696)
Supplement: Supplementary file 1 — Table S1: Parameter estimates for paths from PMQ dimensions to physical activity levels. [file HSR2-9-e72696-s001.docx]

**Supplementary Table 1. Parameter estimates for paths from PMQ dimensions to physical activity levels.**

| Dependent Variable | Predictor | Estimate | SE | 95% CI Lower | 95% CI Upper | β | z | p |
| --- | --- | --- | --- | --- | --- | --- | --- | --- |
| Physical activity levels | Achievement / status | 0.13 | 0.18 | -0.22 | 0.50 | 0.09 | 0.74 | 0.457 |
| Physical activity levels | Team Membership / spirit | -0.03 | 0.11 | -0.26 | 0.19 | -0.03 | -0.30 | 0.763 |
| Physical activity levels | Being Active / skill development | -0.09 | 0.34 | -0.76 | 0.57 | -0.06 | -0.26 | 0.790 |
| Physical activity levels | Competition / excitement | -0.37 | 0.29 | -0.94 | 0.20 | -0.23 | -1.27 | 0.204 |
| Physical activity levels | Fitness | 0.84 | 0.25 | 0.33 | 1.35 | 0.73 | 3.26 | 0.001 |
| Physical activity levels | Energy release | -0.17 | 0.14 | -0.44 | 0.10 | -0.14 | -1.21 | 0.224 |

Note. β = standardized coefficient; SE = standard error; CI = confidence interval.
